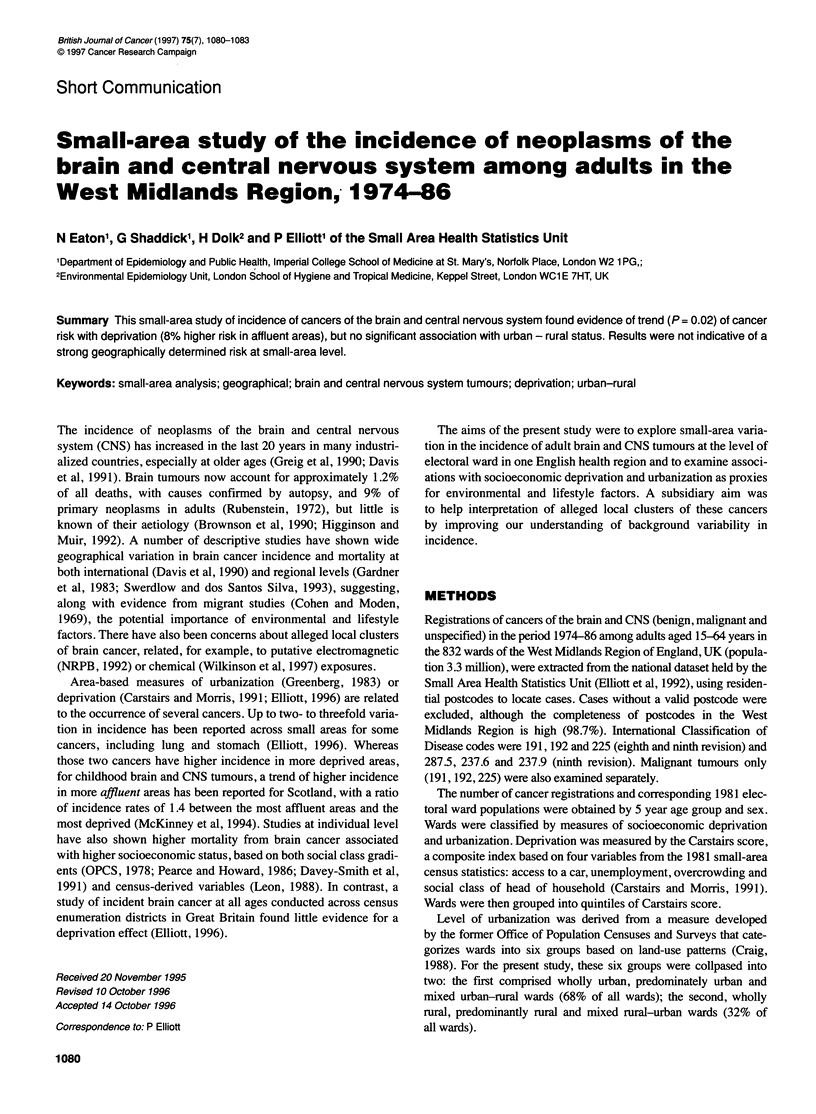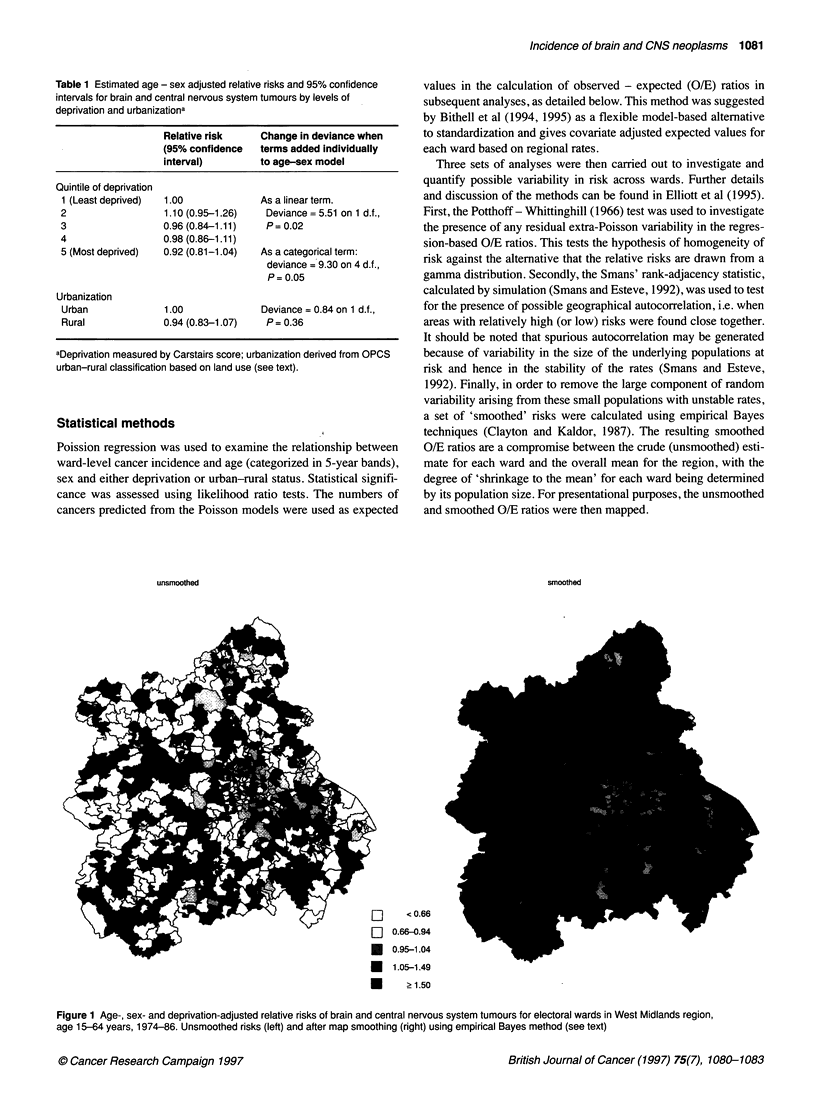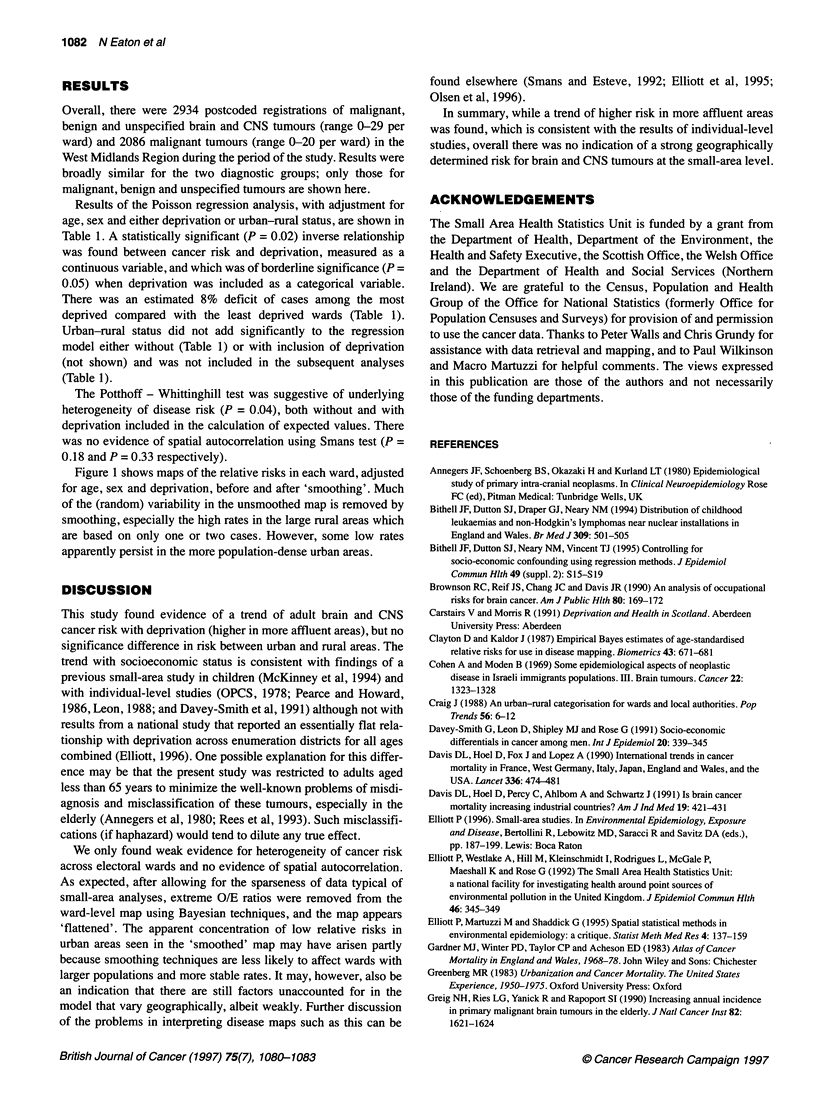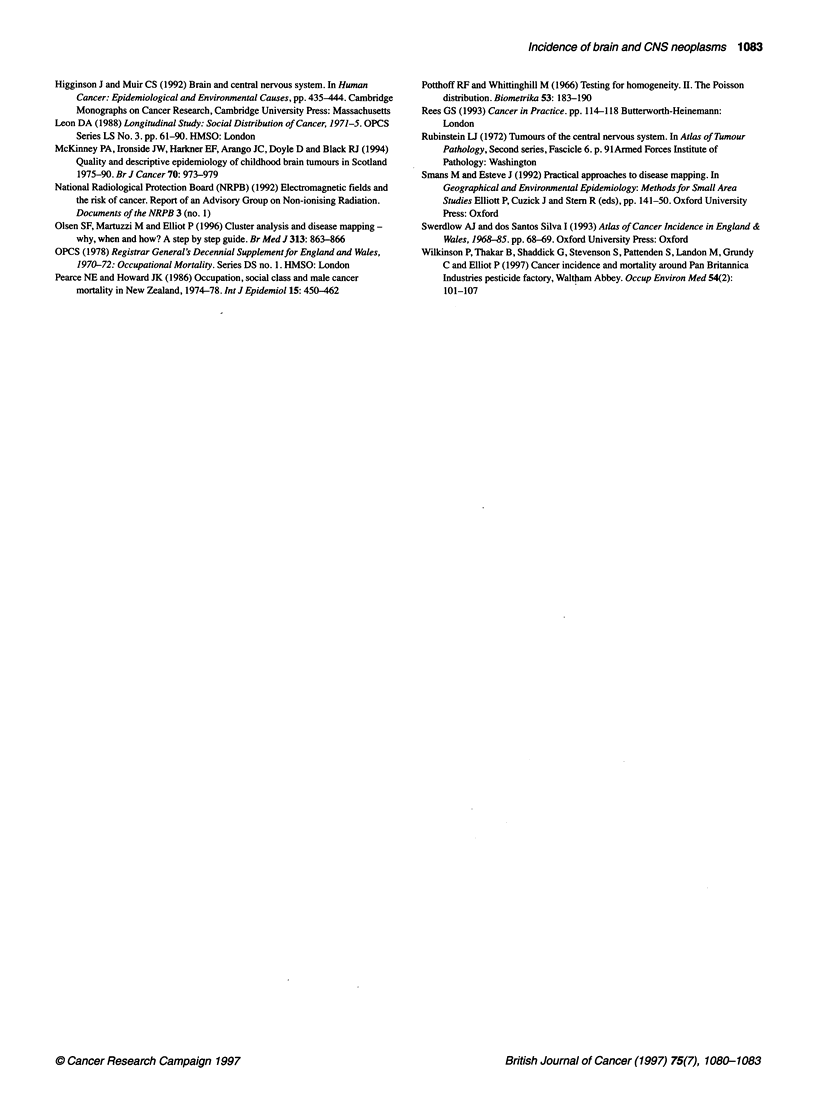# Small-area study of the incidence of neoplasms of the brain and central nervous system among adults in the West Midlands region, 1974-86. Small Area Health Statistics Unit.

**DOI:** 10.1038/bjc.1997.184

**Published:** 1997

**Authors:** N. Eaton, G. Shaddick, H. Dolk, P. Elliott

**Affiliations:** Department of Epidemiology and Public Health, Imperial College School of Medicine at St. Mary's, London, UK.

## Abstract

**Images:**


British Journal of Cancer (1997) 75(7), 1080–1083
© 1997 Cancer Research Campaign

Short Communication

### Small-area study of the incidence of neoplasms of the brain and central nervous system among adults in the West Midlands Region, 1974–86

N Eaton[1], G Shaddick[1], H Dolk[2] and P Elliott[1] of the Small Area Health Statistics Unit

[1]Department of Epidemiology and Public Health, Imperial College School of Medicine at St. Mary's, Norfolk Place, London W2 1PG,;
[2]Environmental Epidemiology Unit, London School of Hygiene and Tropical Medicine, Keppel Street, London WC1E 7HT, UK

**Summary** This small-area study of incidence of cancers of the brain and central nervous system found evidence of trend ($P = 0.02$) of cancer risk with deprivation (8% higher risk in affluent areas), but no significant association with urban – rural status. Results were not indicative of a strong geographically determined risk at small-area level.

**Keywords:** small-area analysis; geographical; brain and central nervous system tumours; deprivation; urban–rural

The incidence of neoplasms of the brain and central nervous system (CNS) has increased in the last 20 years in many industrialized countries, especially at older ages (Greig et al, 1990; Davis et al, 1991). Brain tumours now account for approximately 1.2% of all deaths, with causes confirmed by autopsy, and 9% of primary neoplasms in adults (Rubenstein, 1972), but little is known of their aetiology (Brownson et al, 1990; Higginson and Muir, 1992). A number of descriptive studies have shown wide geographical variation in brain cancer incidence and mortality at both international (Davis et al, 1990) and regional levels (Gardner et al, 1983; Swerdlow and dos Santos Silva, 1993), suggesting, along with evidence from migrant studies (Cohen and Moden, 1969), the potential importance of environmental and lifestyle factors. There have also been concerns about alleged local clusters of brain cancer, related, for example, to putative electromagnetic (NRPB, 1992) or chemical (Wilkinson et al, 1997) exposures.

Area-based measures of urbanization (Greenberg, 1983) or deprivation (Carstairs and Morris, 1991; Elliott, 1996) are related to the occurrence of several cancers. Up to two- to threefold variation in incidence has been reported across small areas for some cancers, including lung and stomach (Elliott, 1996). Whereas those two cancers have higher incidence in more deprived areas, for childhood brain and CNS tumours, a trend of higher incidence in more *affluent* areas has been reported for Scotland, with a ratio of incidence rates of 1.4 between the most affluent areas and the most deprived (McKinney et al, 1994). Studies at individual level have also shown higher mortality from brain cancer associated with higher socioeconomic status, based on both social class gradients (OPCS, 1978; Pearce and Howard, 1986; Davey-Smith et al, 1991) and census-derived variables (Leon, 1988). In contrast, a study of incident brain cancer at all ages conducted across census enumeration districts in Great Britain found little evidence for a deprivation effect (Elliott, 1996).

*Received 20 November 1995*
*Revised 10 October 1996*
*Accepted 14 October 1996*

*Correspondence to:* P Elliott

The aims of the present study were to explore small-area variation in the incidence of adult brain and CNS tumours at the level of electoral ward in one English health region and to examine associations with socioeconomic deprivation and urbanization as proxies for environmental and lifestyle factors. A subsidiary aim was to help interpretation of alleged local clusters of these cancers by improving our understanding of background variability in incidence.

### METHODS

Registrations of cancers of the brain and CNS (benign, malignant and unspecified) in the period 1974–86 among adults aged 15–64 years in the 832 wards of the West Midlands Region of England, UK (population 3.3 million), were extracted from the national dataset held by the Small Area Health Statistics Unit (Elliott et al, 1992), using residential postcodes to locate cases. Cases without a valid postcode were excluded, although the completeness of postcodes in the West Midlands Region is high (98.7%). International Classification of Disease codes were 191, 192 and 225 (eighth and ninth revision) and 287.5, 237.6 and 237.9 (ninth revision). Malignant tumours only (191, 192, 225) were also examined separately.

The number of cancer registrations and corresponding 1981 electoral ward populations were obtained by 5 year age group and sex. Wards were classified by measures of socioeconomic deprivation and urbanization. Deprivation was measured by the Carstairs score, a composite index based on four variables from the 1981 small-area census statistics: access to a car, unemployment, overcrowding and social class of head of household (Carstairs and Morris, 1991). Wards were then grouped into quintiles of Carstairs score.

Level of urbanization was derived from a measure developed by the former Office of Population Censuses and Surveys that categorizes wards into six groups based on land-use patterns (Craig, 1988). For the present study, these six groups were collpased into two: the first comprised wholly urban, predominately urban and mixed urban–rural wards (68% of all wards); the second, wholly rural, predominantly rural and mixed rural–urban wards (32% of all wards).

**Table 1** Estimated age – sex adjusted relative risks and 95% confidence intervals for brain and central nervous system tumours by levels of deprivation and urbanization[a]

| | Relative risk (95% confidence interval) | Change in deviance when terms added individually to age–sex model |
|---|---|---|
| Quintile of deprivation | | |
| 1 (Least deprived) | 1.00 | As a linear term. |
| 2 | 1.10 (0.95–1.26) | Deviance = 5.51 on 1 d.f., |
| 3 | 0.96 (0.84–1.11) | $P = 0.02$ |
| 4 | 0.98 (0.86–1.11) | |
| 5 (Most deprived) | 0.92 (0.81–1.04) | As a categorical term: deviance = 9.30 on 4 d.f., $P = 0.05$ |
| Urbanization | | |
| Urban | 1.00 | Deviance = 0.84 on 1 d.f., |
| Rural | 0.94 (0.83–1.07) | $P = 0.36$ |

[a]Deprivation measured by Carstairs score; urbanization derived from OPCS urban–rural classification based on land use (see text).

### Statistical methods

Poission regression was used to examine the relationship between ward-level cancer incidence and age (categorized in 5-year bands), sex and either deprivation or urban–rural status. Statistical significance was assessed using likelihood ratio tests. The numbers of cancers predicted from the Poisson models were used as expected values in the calculation of observed – expected (O/E) ratios in subsequent analyses, as detailed below. This method was suggested by Bithell et al (1994, 1995) as a flexible model-based alternative to standardization and gives covariate adjusted expected values for each ward based on regional rates.

Three sets of analyses were then carried out to investigate and quantify possible variability in risk across wards. Further details and discussion of the methods can be found in Elliott et al (1995). First, the Potthoff – Whittinghill (1966) test was used to investigate the presence of any residual extra-Poisson variability in the regression-based O/E ratios. This tests the hypothesis of homogeneity of risk against the alternative that the relative risks are drawn from a gamma distribution. Secondly, the Smans' rank-adjacency statistic, calculated by simulation (Smans and Esteve, 1992), was used to test for the presence of possible geographical autocorrelation, i.e. when areas with relatively high (or low) risks were found close together. It should be noted that spurious autocorrelation may be generated because of variability in the size of the underlying populations at risk and hence in the stability of the rates (Smans and Esteve, 1992). Finally, in order to remove the large component of random variability arising from these small populations with unstable rates, a set of 'smoothed' risks were calculated using empirical Bayes techniques (Clayton and Kaldor, 1987). The resulting smoothed O/E ratios are a compromise between the crude (unsmoothed) estimate for each ward and the overall mean for the region, with the degree of 'shrinkage to the mean' for each ward being determined by its population size. For presentational purposes, the unsmoothed and smoothed O/E ratios were then mapped.

**Figure 1** Age-, sex- and deprivation-adjusted relative risks of brain and central nervous system tumours for electoral wards in West Midlands region, age 15–64 years, 1974–86. Unsmoothed risks (left) and after map smoothing (right) using empirical Bayes method (see text)

© Cancer Research Campaign 1997

### RESULTS

Overall, there were 2934 postcoded registrations of malignant, benign and unspecified brain and CNS tumours (range 0–29 per ward) and 2086 malignant tumours (range 0–20 per ward) in the West Midlands Region during the period of the study. Results were broadly similar for the two diagnostic groups; only those for malignant, benign and unspecified tumours are shown here.

Results of the Poisson regression analysis, with adjustment for age, sex and either deprivation or urban–rural status, are shown in Table 1. A statistically significant ($P$ = 0.02) inverse relationship was found between cancer risk and deprivation, measured as a continuous variable, and which was of borderline significance ($P$ = 0.05) when deprivation was included as a categorical variable. There was an estimated 8% deficit of cases among the most deprived compared with the least deprived wards (Table 1). Urban–rural status did not add significantly to the regression model either without (Table 1) or with inclusion of deprivation (not shown) and was not included in the subsequent analyses (Table 1).

The Potthoff – Whittinghill test was suggestive of underlying heterogeneity of disease risk ($P$ = 0.04), both without and with deprivation included in the calculation of expected values. There was no evidence of spatial autocorrelation using Smans test ($P$ = 0.18 and $P$ = 0.33 respectively).

Figure 1 shows maps of the relative risks in each ward, adjusted for age, sex and deprivation, before and after 'smoothing'. Much of the (random) variability in the unsmoothed map is removed by smoothing, especially the high rates in the large rural areas which are based on only one or two cases. However, some low rates apparently persist in the more population-dense urban areas.

### DISCUSSION

This study found evidence of a trend of adult brain and CNS cancer risk with deprivation (higher in more affluent areas), but no significance difference in risk between urban and rural areas. The trend with socioeconomic status is consistent with findings of a previous small-area study in children (McKinney et al, 1994) and with individual-level studies (OPCS, 1978; Pearce and Howard, 1986, Leon, 1988; and Davey-Smith et al, 1991) although not with results from a national study that reported an essentially flat relationship with deprivation across enumeration districts for all ages combined (Elliott, 1996). One possible explanation for this difference may be that the present study was restricted to adults aged less than 65 years to minimize the well-known problems of misdiagnosis and misclassification of these tumours, especially in the elderly (Annegers et al, 1980; Rees et al, 1993). Such misclassifications (if haphazard) would tend to dilute any true effect.

We only found weak evidence for heterogeneity of cancer risk across electoral wards and no evidence of spatial autocorrelation. As expected, after allowing for the sparseness of data typical of small-area analyses, extreme O/E ratios were removed from the ward-level map using Bayesian techniques, and the map appears 'flattened'. The apparent concentration of low relative risks in urban areas seen in the 'smoothed' map may have arisen partly because smoothing techniques are less likely to affect wards with larger populations and more stable rates. It may, however, also be an indication that there are still factors unaccounted for in the model that vary geographically, albeit weakly. Further discussion of the problems in interpreting disease maps such as this can be found elsewhere (Smans and Esteve, 1992; Elliott et al, 1995; Olsen et al, 1996).

In summary, while a trend of higher risk in more affluent areas was found, which is consistent with the results of individual-level studies, overall there was no indication of a strong geographically determined risk for brain and CNS tumours at the small-area level.

### ACKNOWLEDGEMENTS

The Small Area Health Statistics Unit is funded by a grant from the Department of Health, Department of the Environment, the Health and Safety Executive, the Scottish Office, the Welsh Office and the Department of Health and Social Services (Northern Ireland). We are grateful to the Census, Population and Health Group of the Office for National Statistics (formerly Office for Population Censuses and Surveys) for provision of and permission to use the cancer data. Thanks to Peter Walls and Chris Grundy for assistance with data retrieval and mapping, and to Paul Wilkinson and Macro Martuzzi for helpful comments. The views expressed in this publication are those of the authors and not necessarily those of the funding departments.

### REFERENCES

Annegers JF, Schoenberg BS, Okazaki H and Kurland LT (1980) Epidemiological study of primary intra-cranial neoplasms. In *Clinical Neuroepidemiology* Rose FC (ed), Pitman Medical: Tunbridge Wells, UK

Bithell JF, Dutton SJ, Draper GJ, Neary NM (1994) Distribution of childhood leukaemias and non-Hodgkin's lymphomas near nuclear installations in England and Wales. *Br Med J* **309**: 501–505

Bithell JF, Dutton SJ, Neary NM, Vincent TJ (1995) Controlling for socio-economic confounding using regression methods. *J Epidemiol Commun Hlth* **49** (suppl. 2): S15–S19

Brownson RC, Reif JS, Chang JC and Davis JR (1990) An analysis of occupational risks for brain cancer. *Am J Public Hlth* **80**: 169–172

Carstairs V and Morris R (1991) *Deprivation and Health in Scotland*. Aberdeen University Press: Aberdeen

Clayton D and Kaldor J (1987) Empirical Bayes estimates of age-standardised relative risks for use in disease mapping. *Biometrics* **43**: 671–681

Cohen A and Moden B (1969) Some epidemiological aspects of neoplastic disease in Israeli immigrants populations. III. Brain tumours. *Cancer* **22**: 1323–1328

Craig J (1988) An urban–rural categorisation for wards and local authorities. *Pop Trends* **56**: 6–12

Davey-Smith G, Leon D, Shipley MJ and Rose G (1991) Socio-economic differentials in cancer among men. *Int J Epidemiol* **20**: 339–345

Davis DL, Hoel D, Fox J and Lopez A (1990) International trends in cancer mortality in France, West Germany, Italy, Japan, England and Wales, and the USA. *Lancet* **336**: 474–481

Davis DL, Hoel D, Percy C, Ahlbom A and Schwartz J (1991) Is brain cancer mortality increasing industrial countries? *Am J Ind Med* **19**: 421–431

Elliott P (1996). Small-area studies. In *Environmental Epidemiology, Exposure and Disease*, Bertollini R, Lebowitz MD, Saracci R and Savitz DA (eds.), pp. 187–199. Lewis: Boca Raton

Elliott P, Westlake A, Hill M, Kleinschmidt I, Rodrigues L, McGale P, Maeshall K and Rose G (1992) The Small Area Health Statistics Unit: a national facility for investigating health around point sources of environmental pollution in the United Kingdom. *J Epidemiol Commun Hlth* **46**: 345–349

Elliott P, Martuzzi M and Shaddick G (1995) Spatial statistical methods in environmental epidemiology: a critique. *Statist Meth Med Res* **4**: 137–159

Gardner MJ, Winter PD, Taylor CP and Acheson ED (1983) *Atlas of Cancer Mortality in England and Wales, 1968–78*. John Wiley and Sons: Chichester

Greenberg MR (1983) *Urbanization and Cancer Mortality. The United States Experience, 1950–1975*. Oxford University Press: Oxford

Greig NH, Ries LG, Yanick R and Rapoport SI (1990) Increasing annual incidence in primary malignant brain tumours in the elderly. *J Natl Cancer Inst* **82**: 1621–1624

© Cancer Research Campaign 1997

Higginson J and Muir CS (1992) Brain and central nervous system. In *Human Cancer: Epidemiological and Environmental Causes*, pp. 435–444. Cambridge Monographs on Cancer Research, Cambridge University Press: Massachusetts

Leon DA (1988) *Longitudinal Study: Social Distribution of Cancer, 1971–5*. OPCS Series LS No. 3. pp. 61–90. HMSO: London

McKinney PA, Ironside JW, Harkner EF, Arango JC, Doyle D and Black RJ (1994) Quality and descriptive epidemiology of childhood brain tumours in Scotland 1975–90. *Br J Cancer* **70**: 973–979

National Radiological Protection Board (NRPB) (1992) Electromagnetic fields and the risk of cancer. Report of an Advisory Group on Non-ionising Radiation. *Documents of the NRPB* 3 (no. 1)

Olsen SF, Martuzzi M and Elliot P (1996) Cluster analysis and disease mapping – why, when and how? A step by step guide. *Br Med J* **313**: 863–866

OPCS (1978) *Registrar General's Decennial Supplement for England and Wales, 1970–72: Occupational Mortality*. Series DS no. 1. HMSO: London

Pearce NE and Howard JK (1986) Occupation, social class and male cancer mortality in New Zealand, 1974–78. *Int J Epidemiol* **15**: 450–462

Potthoff RF and Whittinghill M (1966) Testing for homogeneity. II. The Poisson distribution. *Biometrika* **53**: 183–190

Rees GS (1993) *Cancer in Practice*. pp. 114–118 Butterworth-Heinemann: London

Rubinstein LJ (1972) Tumours of the central nervous system. In *Atlas of Tumour Pathology*, Second series, Fascicle 6. p. 91Armed Forces Institute of Pathology: Washington

Smans M and Esteve J (1992) Practical approaches to disease mapping. In *Geographical and Environmental Epidemiology: Methods for Small Area Studies* Elliott P, Cuzick J and Stern R (eds), pp. 141–50. Oxford University Press: Oxford

Swerdlow AJ and dos Santos Silva I (1993) *Atlas of Cancer Incidence in England & Wales, 1968–85*. pp. 68–69. Oxford University Press: Oxford

Wilkinson P, Thakar B, Shaddick G, Stevenson S, Pattenden S, Landon M, Grundy C and Elliot P (1997) Cancer incidence and mortality around Pan Britannica Industries pesticide factory, Waltham Abbey. *Occup Environ Med* **54**(2): 101–107

© Cancer Research Campaign 1997